# The Role of *mGluR* Copy Number Variation in Genetic and Environmental Forms of Syndromic Autism Spectrum Disorder

**DOI:** 10.1038/srep19372

**Published:** 2016-01-19

**Authors:** Tara L. Wenger, Charlly Kao, Donna M. McDonald-McGinn, Elaine H. Zackai, Alice Bailey, Robert T. Schultz, Bernice E. Morrow, Beverly S. Emanuel, Hakon Hakonarson

**Affiliations:** 1 Seattle Children’s Hospital, Department of Pediatrics, Seattle, WA 98105 USA; 2 Children’s Hospital of Philadelphia, Department of Pediatrics, Philadelphia, PA 19104 USA; 3 Albert Einstein College of Medicine, Department of Genetics, Bronx, NY 10461 USA

## Abstract

While abnormal signaling mediated through metabotropic glutamate receptor 5 (*mGluR5*) is involved in the pathophysiology of Autism Spectrum Disorder (ASD), Fragile X Syndrome and Tuberous Sclerosis, the role of other *mGluR*s and their associated signaling network genes in syndromic ASD is unknown. This study sought to determine whether *mGluR* Copy Number Variants (CNV’s) were overrepresented in children with syndromic ASD and if *mGluR* “second hit” confers additional risk for ASD in 22q11.2 Deletion Syndrome (22q11DS). To determine whether *mGluR* network CNV’S are enriched in syndromic ASD, we examined microarrays from children with ASD (n = 539). Patient categorization (syndromic vs nonsyndromic) was done via blinded medical chart review in *mGluR* positive and randomly selected *mGluR* negative cases. 11.5% of ASD had *mGluR* CNV’s vs. 3.2% in controls (p < 0.001). Syndromic ASD was more prevalent in children with *mGluR* CNVs (74% vs 16%, p < 0.001). A comparison cohort with 22q11DS (n = 25 with ASD, n = 50 without ASD), all haploinsufficient for mGluR network gene *RANBP1*, were evaluated for “second *mGluR* hits”. 20% with 22q11.2DS + ASD had “second hits” in *mGluR* network genes vs 2% in 22q11.2DS-ASD (p < 0.014). We propose that altered *RANBP1* expression may provide a mechanistic link for several seemingly unrelated genetic and environmental forms of ASD.

Autism Spectrum Disorder (ASD) occurs in approximately 1/88 individuals and is characterized by impairment in social communication and repetitive interests and activities^1^. Approximately 20% of cases occur in the context of an identifiable syndrome^2^. Genetic syndromes with ASD are heterogeneous, including cytogenetically visible chromosomal alterations (e.g. Trisomy 21), microdeletion and microduplication syndromes (e.g. 22q11.2 deletion syndrome [22q11.2DS]); and monogenic disorders (e.g. Fragile X Syndrome [FXS], Tuberous Sclerosis [TS])^3^,^4^,^5^,^6^,^7^,^8^,^9^,^10^,^11^,^12^,^13^. In addition, prenatal exposure to thalidomide, valproic acid, misoprostol, ethanol and maternal rubella infection, have been associated with an elevated risk of ASD^14^,^15^,^16^,^17^,^18^,^19^.

The mechanism for the development of ASD in most forms of idiopathic and syndromic forms of ASD remains elusive. Recently, signaling through metabotropic glutamate receptor 5 (*mGluR5*) as linked to the *mTOR* pathway has been implicated in the development of ASD in FXS and TS^20^. In FXS, abnormal production of Fragile X Mental Retardation Protein (FMRP) removes normal inhibition of signaling through the mGluR pathway. Tuberous Sclerosis leads to over-inhibition of signaling. Auerbach and colleagues demonstrated abnormal synaptic learning and atypical behavior in mouse models of FXS and TS, and reversed these effects by breeding the two strains together – mice harboring both mutations had normal *mGluR* signaling, and learning and behavior that was indistinguishable from control mice^20^. Other studies have demonstrated normalization of learning and behavior in Fragile X mice by administration of an *mGluR5* antagonist^21^,^22^. In addition to elucidating the mechanism for cognitive and behavioral differences in FXS and TS, these studies suggest a promising avenue for pharmacological treatment.

Evidence from recent human studies and animal models suggest that syndromic forms of ASD have behavioral overlap with idiopathic ASD but that there are identifiable behavioral signatures in each disorder^23^. Bruining and colleagues used Support Vector Machine learning to assess behavioral profiles on the Autism Diagnostic Interview in six syndromes with elevated risk of ASD: 22q11.2 Deletion Syndrome, Down’s syndrome, Prader-Willi, supernumerary marker chromosome 15, tuberous sclerosis and Klinefelter syndrome^23^. An identifiable behavioral profile was present within each syndrome for those who met and did not meet criteria for ASD, suggesting a spectrum of severity rather than subgroups of patients with and without ASD in each group. The behavioral signature of children with idiopathic ASD was most similar to children with Tuberous Sclerosis, which the authors propose is due to convergence of many forms of ASD on the *mTOR* pathway.

Our group recently demonstrated that CNVs in the *mGluR* gene network (over 270 genes) occur more frequently in children with ASD than in controls^24^. Other recent studies have proposed that phenotypic differences might be seen in children harboring mutations affecting genes associated with the *mGluR* gene network, including those with identifiable syndromes^23^,^25^. One limitation of these prior studies is that careful identification of children who may be more likely to have a syndromic form of ASD is limited. Approximately 20% of children with ASD are suspected to have an underlying genetic syndrome^2^. For this reason, the American College of Medical Genetics recommends that all children with an ASD receive a genome-wide microarray and undergo an evaluation by a clinical geneticist. However, to this date, only a small fraction of children with ASD have been evaluated by a clinical geneticist. Among our patient population through the Center for Autism Research, chart review of over 500 children with ASD evaluated by the center revealed that less than 10% had received any prior genetic testing (data not shown). In addition, as many disparate CNVs, distinct genetic syndromes and environmental exposure syndromes have an elevated rate of ASD in addition to birth defects. Birth defects are not reported to be more frequent in children with idiopathic ASD. In this way, the presence of birth defects with ASD may be a marker that identifies children with a syndromic form of ASD that has yet to be clinically recognized.

For this reason, this study sought to closely investigate whether children with ASD identified to have *mGluR* CNVs would have features suggestive of Syndromic ASD (e.g. birth defects) at a higher rate than children with ASD and no *mGluR* network CNVs. Further, to investigate the role of *mGluR* network CNVs in the development of ASD in vulnerable syndromes, we studied children with 22q11.2 Deletion Syndrome, who all have deletion of the *mGluR* gene *RANBP1.* We identified children with 22q11.2DS (n = 75), including those with ASD (n = 25) and without ASD (n = 50), and looked for the rate of “second hits” in the *mGluR* network to determine if additional disease risk is incurred for ASD with double “hits” to the *mGluR* network.

## Results

### Syndromic features common in children with ASD with mGluR CNVs

**Figure 1:**
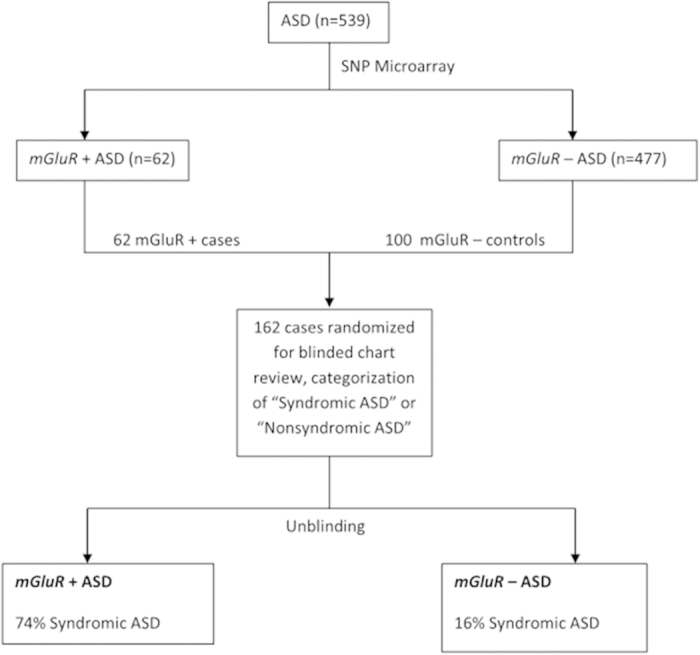
Schematic of study design. Children with ASD and gene changes in *mGluR* network (*mGluR* + ASD) were more likely to have Syndromic ASD compared to children with ASD without abnormalities of *mGluR* network genes (*mGluR* – ASD).p < 0.0001. This remained significant after limiting the analysis to patients in the *mGluR*+ and *mGlurR*- groups with comparable overall size and number of CNV’s (p < 0.0001).

We focused our analysis on CNVs in the *mGluR* gene signaling network previously shown to be enriched in ASD (for detailed description of justification and methods see Hadley *et al*.^24^). Children with ASD and *mGluR* CNVs were more likely to have syndromic features (e.g. birth defects, documentation of suspicion for genetic disorder based on dysmorphic features or uncommon medical problems, or clinical diagnosis of genetic syndrome) than children with ASD without *mGluR* CNVs (74% of *mGluR* + classified as “Syndromic” vs. 16% of *mGluR* – ASD; p < 0.0001). See Fig. 1. Many of the *mGluR* CNVs in patients with syndromic ASD were included in larger clinically significant CNVs, but others occurred in smaller deleted or duplicated regions comparable in size to CNV’s in the *mGluR*- group (See supplementary table 1). As *mGluR* network genes are present in the 22q11.2 region (*RANBP1*) and on chromosome 21 (*APP GRIK1 MX1 PCBP3 SETD4*), patients with ASD in the presence of 22q11.2DS, 22q11.2DupS or Trisomy 21 accounted for approximately one third of the patients with Syndromic ASD + *mGluR* network changes. The remainder of observed cytogenetic changes had individual non-overlapping deletions or duplications. As a second analytic step, all patients with very large deletions or duplications were excluded, and the mGluR+ and mGluR- groups were matched for overall CNV size and burdon. Comparison test for CNV number and burdon was Student’s T-test, two tailed, with nonequivalent variances. Although there was no significant residual difference in the CNV size (p = 0.18) or numbers (p = 0.11) between the *mGluR+* and *mGluR−* groups, there was a highly significant difference in the presence of syndromic features between the mGluR+ and mGluR− groups (p < 0.0001).

### Second hit in mGluR gene network associated with increased risk of ASD in 22q11.2 Deletion Syndrome

As a comparison cohort, data from children with 22q11.2DS with ASD (n = 25) and without ASD (n = 50) who had completed high density microarray evaluation (either Affymetrix 6.0, Illumina HH550K, and Illumina 610Q) and clinical developmental assessments (as enrolled through a parallel study, approved by the Children’s Hospital of Philadelphia Institutional Review Board, IRB 07-005352) were examined for the presence of a second *mGluR* network hit outside of the 22q11.2 region, using the same protocol as in Hadley *et al*. (2014). This ensured that the same criteria were used to identify an *mGluR* CNV in the ASD group and in the second cohort of children with 22q11.2DS. “Second hits”, deletions or duplications of an *mGluR* network gene outside of the 22q11.2 region, were found in 20% (5/25) of patients with ASD and only 2% (1/50) without ASD (p < 0.014). For complete list of second hits, see Table 1.

## Discussion

Prior studies have demonstrated that abnormal signaling (either too much or too little) through *mGluR5* could be the basis for abnormal neural development (and possibly ASD) in FXS and TS. Our data suggest that derangement of the *mGluR* network may be responsible for increased rates of ASD seen in cytogenetically distinct forms of syndromic ASD. *mGluR* network genes are found in the 22q11.2 region as well as on Chromosome 21, which may be involved in the increased prevalence of ASD in both Down Syndrome and 22q11.2DS. However, all patients with Trisomy 21 or 22q11.2DS harbor the change in the *mGluR* network suggesting a second hit outside of the region may be necessary for expression of the ASD phenotype. Here, we demonstrate second hits as significantly contributing to the development of ASD in 22q11.2DS. Animal studies support abnormal neural development in *Ranbp1* (−/−) mice^26^.

The 22q11.2DS is the most common microdeletion syndrome in humans, occurring in 1 in 2–4,000 individuals. The typical deletion spans approximately 3 Mb and includes approximately 45 genes, causing a variety of medical and behavioral disorders (Table 1)^27^,^28^,^29^,^30^. ASD occurs in approximately 20%, and psychosis in approximately 25%^5^,^9^,^27^,^28^,^30^. For this study we chose 22q11.2DS as a population for second hit evaluation for several reasons: 1) The authors are involved in a parallel study focusing on neurodevelopmental outcomes in children with 22q11.2 related disorders has ensured that there is thorough documentation of developmental assessments, which have all been done by a small group of developmental pediatricians and psychologists skilled in the diagnosis of ASD, making us confident in the designations of ASD and no ASD for our analysis; 2) Our hospital has a large 22q11.2 clinic that has served over 1,000 patients with 22q11.2 related disorders, so all providers are skilled in the recognition and treatment of 22q11.2 associated medical and developmental issues; 3) Individuals with 22q11.2DS typically have normal intelligence or mild intellectual disability, making their assessments for ASD less challenging than Trisomy 21, in which more profound intellectual disability is the norm; 4) The rate of ASD is high enough that we were able to confidently select 50 patients with documentation of no concerns for ASD and 25 patients with diagnoses of ASD by an experienced examiner. The genetic cause for ASD within the 22q11.2 region is unknown. We hypothesize in this study that the risk may be due to a combination of haploinsufficiency in the mGluR associated gene, *RANBP1*, together with second hits in the mGluR network for a portion of patients with 22q11.2DS. It is possible that second hits in additional gene networks that were not evaluated as part of this study also have a role in the development of ASD in 22q11.2DS.

In order to further understand whether *RANBP1* could play a role in the development of ASD in 22q11.2DS, we examined data from animal models, *in vitro* studies and human teratogenic exposures in which a high rate of ASD has been described. Paronett *et al*.^26^ recently reported the first animal model of *Ranbp1* (−/−) mice. These mice had abnormal cortical development, including decreases in cortical thickness and number of neurons. This is particularly important because the 22q11.2 region contains approximately 40 genes, and this finding implicates *Ranbp1* in brain development.

Based on our group’s prior work^24^ as well as the present study, we hypothesized that haploinsufficiency of the *mGluR* network gene in the 22q11.2 region, *RANBP1*, could confer risk for ASD in 22q11.2DS. Of note, five environmental agents have been shown to increase the rate of ASD with prenatal exposure, including thalidomide and valproic acid, which have been linked to the *mGluR* network or *RANBP1*^26^,^33^,^34^,^35^,^36^,^37^. Meganathan and colleagues hypothesized thalidomide embryopathy was mediated via effects of RANPB1 using a human embryonic stem cell model^32^. Supposing that ASD in thalidomide embryopathy and fetal valproate syndrome could be linked to a common mechanism with 22q11.2DS, a similar pattern of birth defects would be anticipated. A supplementary table lists the published birth defects in thalidomide embryopathy and fetal valproate syndrome compared to our patient cohort with 22q11.2DS. Every birth defect seen in either thalidomide embryopathy or fetal valproate syndrome has been observed in our cohort of patients with 22q11.2DS. Additional medical conditions are present in 22q11.2DS, which includes deletion of dozens of genes not hypothesized to be affected by exposure to thalidomide or valproate.

In the present study we found derangement of genes in the *mGluR* network at a high rate in patients with different forms of Syndromic ASD, including 22q11.2DS, Trisomy 21 and a large number of other seemingly-unrelated chromosomal alterations. Moreover, among children with 22q11.2DS, the presence of a “second hit” in the *mGluR* network was identified in 20% of children with ASD, and only 2% of those without ASD (p < 0.014). In children with 22q11.2DS, there was decreased expression in *RANBP1* (data not shown).

Taken together, these data suggest that dysregulation of the *mGluR* network is a possible permissive factor that increases the propensity to develop an ASD. The striking increase in prevalence of ASD with a CNV affecting a second gene in the network suggests perturbations of *mGluR* signaling at multiple points is necessary. It is important to note that CNVs may only represent a fraction of changes in *mGluR* network genes, as this study did not include assessment of sequence variations, and these findings may therefore represent the “tip of the iceberg”. While perturbation of the *mGluR* network appears to confer risk of ASD, additional genetic or environmental stressors are likely necessary for an individual child to develop ASD.

Striking similarities exist in the profiles of birth defects and elevated rates of Autism Spectrum Disorder seen in 22q11.2 Deletion Syndrome, Fetal Valproate Syndrome and Thalidomide Embryopathy. As thalidomide and VPA both cause decreased expression of *RANBP1* mRNA, mimicking haploinsufficiency of the gene in 22q11.2 Deletion Syndrome, it is plausible that it could be involved in the common teratogenic profile across syndromes. Moreover, results from a *Ranbp1* knockout mouse model from Paronett *et al*.^26^ are also supportive of our hypothesis of the importance of *RANBP1*in the neurological consequences of 22q11.2DS and prenatal exposures affecting expression of *RANBP1*. In these studies, Ranbp1 (−/−) homozygotes, proliferation of the basal progenitor pool in the cortex is disrupted, leading to a dramatic reduction in cortical thickness and substantially fewer neurons in the perinatal cortex. The changes resulting from loss of RANBP1 function parallel that seen in mice with the larger 22q11.2 Deletion, suggesting that haploinsufficiency of Ranbp1 may contribute to the disruption of cortical circuitry in 22q11DS. Future studies, addressing the neurodevelopmental phenotype of mice with haploinsufficiency of *Ranbp1* are anticipated to help elucidate the mechanism by which alterations of the *mGluR* pathway leads to increased risk of ASD.

## Methods

### Participants

Phenotypic data for patients with ASD as reported on parental health questionnaires from our biorepository (n = 6452), which includes samples from inside and outside our institution, were evaluated to identify patients who received clinical assessment at the Children’s Hospital of Philadelphia and agreed to Electronic Health Record chart review. Medical records were available for 539 of these patients. Children were recruited for inclusion in the general Center for Applied Genomics biorepository when they were getting blood drawn for another purpose at The Children’s Hospital of Philadelphia, so there is an overrepresentation of children with at least one medical problem in this patient cohort. DNA from 539 ASD cases were selected for further phenotypic and genotypic analysis. The parents of all patients gave informed consent for participation in the study, which was performed in accordance with relevant guidelines and regulations approved by the Institutional Review Board at the Children’s Hospital of Philadelphia (IRB 06-004886).

### Chart review

Subject selection and randomization process: All patients with an *mGluR* CNV (n = 62) and 100 patients without *mGluR* CNV were randomly selected for chart review. This procedure was selected to ensure that all patients with *mGluR* CNV received detailed chart review with an adequately sized comparison cohort. A three step process was done to ensure blinded chart review. The selection of the 162 charts was done by a geneticist with access to CNV data but without access to the Electronic Health Record (CK). Another author who had no access to CNV data nor the Electronic Health Record blinded and randomized the patient ID’s (RTS). Finally, a physician with access to the Electronic Health Record but blinded to *mGluR* status (TLW) reviewed charts for documentation of ASD diagnosis and presence of other medical comorbidities.

### Medical chart review

ASD: Charts were reviewed to confirm a diagnosis of ASD and also to determine medical comorbidities for each patient. Diagnosis of ASD was confirmed in the chart, but as this was a retrospective chart review, gold-standard research instruments (e.g. Autism Diagnostic Interview; Autism Diagnostic Observation Schedule) were not always available for review, while both parental intake surveys and Electronic Medical Records from the Children’s Hospital of Philadelphia referenced ASD evaluations. Patient categorization of “Syndromic” vs “Nonsyndromic” ASD was based on the presence of additional birth defects and documentation of dysmorphic features in the medical record. For complete description of this process, see online supplemental methods.

### Genotyping and CNVs

A brief description of methods for CNV calling is included in supplemental online material. For a more detailed justification and description of methods related to CNV identification and list of genes included in *mGluR* network, please refer to Hadley *et al* (2014)^24^.

## Additional Information

**How to cite this article**: Wenger, T. L. *et al*. The Role of *mGluR* Copy Number Variation in Genetic and Environmental Forms of Syndromic Autism Spectrum Disorder. *Sci. Rep.* **6**, 19372; doi: 10.1038/srep19372 (2016).

## Supplementary Material

Supplemental Methods

Supplementary Table 1

Supplementary Table 2

## Figures and Tables

**Table 1:** Second hits in mGluR network genes in patients with 22q11.2 Deletion Syndrome.

**22q11.2 with mGluR second hits**	**Classic 22q11.2 deletion**	**Autism**	**Size**	**mGluR genes**
1	Yes	Yes	869 kb duplication	GRM3
2	Yes	Yes	145 kb deletion	HRAS
3	Yes	Yes	249 kb deletion	HRAS
4	Yes	Yes	20.8 kb deletion	NRXN1
5	Yes	Yes	9.5 kb duplication	GNB2L1
6	Yes	No	65.9 kb duplication	MKNK2

## References

[b1] Baio J. . Prevalence of Autism Spectrum Disorders — Autism and Developmental Disabilities Monitoring Network, 14 Sites, United States, 2008. at http://www.cdc.gov/mmwr/preview/mmwrhtml/ss6103a1.htm?s_cid=ss6103a1_w Date of access 6/2/2015. 22456193

[b2] Gurrieri F. Working up autism: the practical role of medical genetics. in American Journal of Medical Genetics Part C: Seminars in Medical Genetics 160, 104–110 (2012). 10.1002/ajmg.c.31326 22499531

[b3] Christian S. L. . Novel submicroscopic chromosomal abnormalities detected in autism spectrum disorder. Biol. Psychiatry 63, 1111 (2008). 18374305 10.1016/j.biopsych.2008.01.009 PMC2440346

[b4] Gillberg C. Chromosomal disorders and autism. J. Autism Dev. Disord. 28, 415–425 (1998). 9813777 10.1023/a:1026004505764

[b5] Fine S. E. . Autism spectrum disorders and symptoms in children with molecularly confirmed 22q11. 2 deletion syndrome. J. Autism Dev. Disord. 35, 461–470 (2005). 16134031 10.1007/s10803-005-5036-9 PMC2814423

[b6] Lo-Castro A. . Association of syndromic mental retardation and autism with 22q11. 2 duplication. Neuropediatrics 40, 137–140 (2009). 20020400 10.1055/s-0029-1237724

[b7] Motavalli Mukaddes N. & Herguner S. Autistic disorder and 22q11. 2 duplication. World J. Biol. Psychiatry 8, 127–130 (2007). 17455106 10.1080/15622970601026701

[b8] Ramelli G. P. . Microduplication 22q11. 2 in a child with autism spectrum disorder: clinical and genetic study. Dev. Med. Child Neurol. 50, 953–955 (2008). 19046189 10.1111/j.1469-8749.2008.03048.x

[b9] Vorstman J. A. . The 22q11. 2 deletion in children: high rate of autistic disorders and early onset of psychotic symptoms. J. Am. Acad. Child Adolesc. Psychiatry 45, 1104–1113 (2006). 16926618 10.1097/01.chi.0000228131.56956.c1

[b10] Mazzocco M. M., Kates W. R., Baumgardner T. L., Freund L. S. & Reiss A. L. Autistic behaviors among girls with fragile X syndrome. J. Autism Dev. Disord. 27, 415–435 (1997). 9261667 10.1023/a:1025857422026

[b11] Brown W. T. . Fragile X and autism: a multicenter survey. Am. J. Med. Genet. 23, 341–352 (1986). 3513570 10.1002/ajmg.1320230126

[b12] Gillberg J. C., Gillberg C. & Ahlsén G. Autistic behaviour and attention deficits in tuberous sclerosis: a population-based study. Dev. Med. Child Neurol. 36, 50–56 (1994). 8132114 10.1111/j.1469-8749.1994.tb11765.x

[b13] Jeste S. S., Sahin M., Bolton P., Ploubidis G. B. & Humphrey A. Characterization of autism in young children with tuberous sclerosis complex. J. Child Neurol. 23, 520–525 (2008). 18160549 10.1177/0883073807309788

[b14] Nanson J. L. Autism in fetal alcohol syndrome: a report of six cases. Alcohol. Clin. Exp. Res. 16, 558–565 (1992). 1626656 10.1111/j.1530-0277.1992.tb01417.x

[b15] Bandim J. M., Ventura L. O., Miller M. T., Almeida H. C. & Costa A. E. S. Autism and Möbius sequence: an exploratory study of children in northeastern Brazil. Arq. Neuropsiquiatr. 61, 181–185 (2003). 12806493 10.1590/s0004-282x2003000200004

[b16] Strömland K., Nordin V., Miller M., Akerström B. & Gillberg C. Autism in thalidomide embryopathy: a population study. Dev. Med. Child Neurol. 36, 351–356 (1994). 8157157 10.1111/j.1469-8749.1994.tb11856.x

[b17] Chess S. Autism in children with congenital rubella. *J. Autism Child. Schizophr* . 1, 33–47 (1971). 5172438 10.1007/BF01537741

[b18] Chess S. Follow-up report on autism in congenital rubella. J. Autism Child. Schizophr . 7, 69–81 (1977). 576606 10.1007/BF01531116

[b19] Christensen J. . Prenatal Valproate Exposure and Risk of Autism Spectrum Disorders and Childhood Autism. JAMA 309, 1696–1703 (2013). 23613074 10.1001/jama.2013.2270 PMC4511955

[b20] Auerbach B. D., Osterweil E. K. & Bear M. F. Mutations causing syndromic autism define an axis of synaptic pathophysiology. Nature 480, 63–68 (2011). 22113615 10.1038/nature10658 PMC3228874

[b21] Thomas A. M., Bui N., Perkins J. R., Yuva-Paylor L. A. & Paylor R. Group I metabotropic glutamate receptor antagonists alter select behaviors in a mouse model for fragile X syndrome. Psychopharmacology (Berl.) 219, 47–58 (2012). 21656124 10.1007/s00213-011-2375-4

[b22] Choi C. H. . Pharmacological reversal of synaptic plasticity deficits in the mouse model of fragile X syndrome by group II mGluR antagonist or lithium treatment. Brain Res. 1380, 106–119 (2011). 21078304 10.1016/j.brainres.2010.11.032 PMC3050427

[b23] Bruining H. . Behavioral signatures related to genetic disorders in autism. Mol. Autism 5, 1–12 (2014). 24517317 10.1186/2040-2392-5-11 PMC3936826

[b24] Hadley D. . The impact of the metabotropic glutamate receptor and other gene family interaction networks on autism. *Nat Commun*. 5, 40174 (2014). doi: 10.1038/ncomms5074. PMC4059929 24927284

[b25] Gai X. . Rare structural variation of synapse and neurotransmission genes in autism. Mol. Psychiatry 17, 402–411 (2011). 21358714 10.1038/mp.2011.10 PMC3314176

[b26] Paronett E. M., Meechan D., Karpinsky B. A., LaMantia A.-S. & Maynard T. M. Ranbp1, Deleted in DiGeorge/22q11.2 Deletion Syndrome, is a Microcephaly Gene That Selectively Disrupts Layer 2/3 Cortical Projection Neuron Generation. *Cereb. Cortex*. (2014). doi: 10.1093/cercor/bhu28537. PMC4585528 25452572

[b27] Bassett A. S. . Practical Guidelines for Managing Patients with 22q11.2 Deletion Syndrome. J. Pediatr. 159, 332–9. e1 (2011). 21570089 10.1016/j.jpeds.2011.02.039 PMC3197829

[b28] Goodship J., Cross I., LiLing J. & Wren C. A population study of chromosome 22q11 deletions in infancy. Arch. Dis. Child. 79, 348–351 (1998). 9875047 10.1136/adc.79.4.348 PMC1717723

[b29] Du Montcel S. T., Mendizabai H., Ayme S., Levy A. & Philip N. Prevalence of 22q11 microdeletion. J. Med. Genet. 33, 719 (1996). 10.1136/jmg.33.8.719 PMC1050716 8863171

[b30] Oskarsdottir S., Vujic M. & Fasth A. Incidence and prevalence of the 22q11 deletion syndrome: a population-based study in Western Sweden. Arch. Dis. Child. 89, 148–151 (2004). 14736631 10.1136/adc.2003.026880 PMC1719787

[b31] Mehta M. V., Gandal M. J. & Siegel S. J. mGluR5-antagonist mediated reversal of elevated stereotyped, repetitive behaviors in the VPA model of autism. PloS One 6, e26077 (2011). 22016815 10.1371/journal.pone.0026077 PMC3189241

[b32] Meganathan K. . Identification of thalidomide-specific transcriptomics and proteomics signatures during differentiation of human embryonic stem cells. PLoS One 7, e44228 (2012). 22952932 10.1371/journal.pone.0044228 PMC3429450

[b33] Rasalam A. D. . Characteristics of fetal anticonvulsant syndrome associated autistic disorder. Dev. Med. Child Neurol. 47, 551–555 (2005). 16108456 10.1017/s0012162205001076

[b34] Moore S. J. . A clinical study of 57 children with fetal anticonvulsant syndromes. J. Med. Genet. 37, 489–497 (2000). 10882750 10.1136/jmg.37.7.489 PMC1734633

[b35] Ingram J. L., Peckham S. M., Tisdale B. & Rodier P. M. Prenatal exposure of rats to valproic acid reproduces the cerebellar anomalies associated with autism. Neurotoxicol. Teratol. 22, 319–324 (2000). 10840175 10.1016/s0892-0362(99)00083-5

[b36] Rodier P. M., Ingram J. L., Tisdale B., Nelson S. & Romano J. Embryological origin for autism: developmental anomalies of the cranial nerve motor nuclei. J. Comp. Neurol. 370, 247–261 (1996). 8808733 10.1002/(SICI)1096-9861(19960624)370:2<247::AID-CNE8>3.0.CO;2-2

[b37] Tomasz Schneider R. P. Behavioral alterations in rats prenatally exposed to valproic acid: animal model of autism. Neuropsychopharmacology 30, 80–89 (2004). 15238991 10.1038/sj.npp.1300518

