# Supplementary material for: The Role of mGluR Copy Number Variation in Genetic and Environmental Forms of Syndromic Autism Spectrum Disorder
Source: Sci Rep. 2016 Jan 19;6:19372. doi: 10.1038/srep19372 (PMC4726047; doi:10.1038/srep19372)
Supplement: Supplemental Methods [file srep19372-s1.doc]

**The Role of *mGluR* Copy Number Variation in Genetic and Environmental Forms of Syndromic Autism Spectrum Disorder**

**Tara L. Wenger^1, 2^, Charlly Kao^2^, Donna M. McDonald-McGinn, M.S.^2^, Elaine H. Zackai^2^, Alice Bailey^2^, Robert T. Schultz^2^, Bernice E. Morrow^3^, Beverly S. Emanuel^2^, Hakon Hakonarson^2*^**

^1^Seattle Children’s Hospital, Department of Pediatrics, Seattle, WA 98105 USA

^2^Children’s Hospital of Philadelphia, Department of Pediatrics, Philadelphia, PA 19104 USA

^3^Albert Einstein College of Medicine, Department of Genetics, Bronx, NY 10461 USA

*hakonarson@email.chop.edu

**Supplemental Methods**

**Medical comorbidities**

As discussed above, this study sought to identify cases of syndromic ASD in a cohort with a low rate of having been evaluated by a clinical geneticist. Children with idiopathic ASD should not have an elevated rate of birth defects as compared to the general population. In contrast, children with ASD in the context of several genetic syndromes and environmental exposure syndromes have elevated rates of birth defects. For this reason, charts were reviewed by a blinded physician for medical comorbidities suggestive of an underlying genetic diagnosis. Structural birth defects, genetic testing and medical conditions were recorded for each patient. Cases were categorized as “Syndromic ASD” if they had ASD and presence of a medical condition/structural birth defect (e.g. cleft palate) that occurs in less than 1% of the general population. This criteria was established to define a subset of patients whose ASD and other medical problems would be highly unlikely to occur coincidentally – with a baseline rate of ASD at 1/88 and a medical condition that occurs in <1% of the general population, the compound likelihood of both occurring by chance would be approximately 0.001%.

See Figure 1. For additional details on the methods, please see online supplemental materials.

**Genotyping Arrays and CNV Calling**

DNA from subjects with ASD were each genotyped on the Human610-Quad or HumanHap550 SNP arrays from Illumina. For 22q11 DS cohorts, subjects were typed either on Illumina SNP arrays (Human610-Quad v1.0 or HumanHap550) or Affymetrix 6.0 SNP arrays. Clustering and SNP calling was performed using GenomeStudio (Illumina) to generate normalized intensity (i.e. Log-R ratio, or LRR) and B-allele frequencies (BAF). CNV calling was performed using the PennCNV algorithm [PMID: 17921354] following waviness correction [PMID: 18784189]. In brief, PennCNV uses a hidden Markov model (HMM) that incorporates information from LRR, BAF, as well as features of the array (e.g. distance between neighboring SNPs) to detect CNVs.

**CNV Quality Control**

Samples with SNP arrays of poor quality were excluded from CNV calling, since typically the proportion of false positives increases considerably for these samples. Those samples where the genotyping call rate > 96%, standard deviation of LRR (LRR sd) < 0.4, GC-wave factor (GCWF) is between -0.2 and 0.2 after waviness correction, and total number of CNV calls for the sample < 100 were included in analysis.

**CNV Annotation**

For syndromic ASD regions, genomic coordinates were those described by Betancur [PMID: 21129364].  The GRM/mGluR network generated by Cytoscape from the Human Interactome database was described by Elia et al. [PMID: 22138692] using UCSC Genome Browser definitions for gene coordinates (UCSC genes). This network from Cytoscape was used to define mGluR+ vs. mGluR- subsets. For 22q11 DS cohort analysis, additional GRM/mGluR network genes were identified based on 1^st^ degree interaction network of the eight GRM genes using the program Ingenuity Pathway Analysis (Ingenuity Systems Inc./Qiagen; Redwood City, CA) as well as the genes encoding the group I mGluR signaling pathway described in Kelleher et al. [PMID: 22558107]. CNV calls were analyzed for overlap to known syndromic regions and GRM network genes. All syndromic aberrations detected by clinical cytogenetic laboratory testing were confirmed on corresponding SNP arrays.
