# Supplementary material for: The Role of mGluR Copy Number Variation in Genetic and Environmental Forms of Syndromic Autism Spectrum Disorder
Source: Sci Rep. 2016 Jan 19;6:19372. doi: 10.1038/srep19372 (PMC4726047; doi:10.1038/srep19372)
Supplement: Supplementary Table 1 [file srep19372-s2.doc]

**The Role of *mGluR* Copy Number Variation in Genetic and Environmental Forms of Syndromic Autism Spectrum Disorder**

**Tara L. Wenger^1, 2^, Charlly Kao^2^, Donna M. McDonald-McGinn, M.S.^2^, Elaine H. Zackai^2^, Alice Bailey^2^, Robert T. Schultz^2^, Bernice E. Morrow^3^, Beverly S. Emanuel^2^, Hakon Hakonarson^2*^**

^1^Seattle Children’s Hospital, Department of Pediatrics, Seattle, WA 98105 USA

^2^Children’s Hospital of Philadelphia, Department of Pediatrics, Philadelphia, PA 19104 USA

^3^Albert Einstein College of Medicine, Department of Genetics, Bronx, NY 10461 USA

*hakonarson@email.chop.edu

**Supplementary Table 1. Prior clinical diagnoses, size and coordinates of CNV’s in *mGluR* positive patients with ASD**

| Prior clinical diagnosis of genetic syndrome | mGluR network gene | Deletion or duplication | CNV coordinates | Size | # of SNPs | StartSNP | EndSNP |
| --- | --- | --- | --- | --- | --- | --- | --- |
| Trisomy 21 | APP GRIK1 MX1 PCBP3 SETD4 | Trisomy | chr21 | N/A | N/A | N/A | N/A |
| Trisomy 21 | APP GRIK1 MX1 PCBP3 SETD4 | Trisomy | chr21 | N/A | N/A | N/A | N/A |
| Trisomy 21 | APP GRIK1 MX1 PCBP3 SETD4 | Trisomy | chr21 | N/A | N/A | N/A | N/A |
| Trisomy 21 | APP GRIK1 MX1 PCBP3 SETD4 | Trisomy | chr21 | N/A | N/A | N/A | N/A |
| Trisomy 21 | APP GRIK1 MX1 PCBP3 SETD4 | Trisomy | chr21 | N/A | N/A | N/A | N/A |
| Trisomy 21 | APP GRIK1 MX1 PCBP3 SETD4 | Trisomy | chr21 | N/A | N/A | N/A | N/A |
| Trisomy 21 | APP GRIK1 MX1 PCBP3 SETD4 | Trisomy | chr21 | N/A | N/A | N/A | N/A |
| Trisomy 21 | APP GRIK1 MX1 PCBP3 SETD4 | Trisomy | chr21 | N/A | N/A | N/A | N/A |
| Trisomy 21 | APP GRIK1 MX1 PCBP3 SETD4 | Trisomy | chr21 | N/A | N/A | N/A | N/A |
| Trisomy 21 | APP GRIK1 MX1 PCBP3 SETD4 | Trisomy | chr21 | N/A | N/A | N/A | N/A |
| Trisomy 21 | APP GRIK1 MX1 PCBP3 SETD4 | Trisomy | chr21 | N/A | N/A | N/A | N/A |
| Turners w one abnormal X, Inv DupX q21.2q24 | FMR1 FLNA NLGN4X NLGN3 | Complex | chrX | N/A | N/A | N/A | N/A |
| Monosomy 7, +mar der(7) | ADCY1 GRM8 | Complex | chr7 | N/A | N/A | N/A | N/A |
| Unbalanced karyotype from complex 3-way translocation, dup of 47 Mb in 3q24q29, Del of 2.87 Mb wihtin 4q35.2 | TNIK PIK3CA | Duplication | chr3:148718395-195285633 | 46,567,239 | 8326 | rs4681476 | rs823504 |
| Cri du chat syndrome | LRP2BP MTNR1A containing LRP2BP MTNR1A | Deletion | chr4:163707357-189351268 | 25,643,912 | 5210 | rs10007190 | rs869570 |
| n/a | MYO6 | Deletion | chr6:62259205-76876104 | 14,616,900 | 2399 | rs1336353 | rs6900948 |
| n/a | DRD3 | Deletion | chr3:106458422-115916171 | 9,457,750 | 1596 | rs1915327 | rs6779886 |
| n/a | ITGB1 | Duplication | chr10:28761159-36346759 | 7,585,601 | 1549 | rs2790441 | rs11010390 |
| Deletion of 6.53 Mb region within 21q22.2q22.3; Mosaic deletion of 1.06 Mb region within 21q22.2 | PCBP3 MX1 | Deletion | chr21:41592144-48084989 | 6,492,846 | 1841 | rs11908752 | rs2839378 |
| Complex karyotype, insertion of portion of 9 into 5, interstitial deletion of 12q | DCN | Deletion | chr12:87447757-92982598 | 5,534,842 | 773 | rs12422524 | rs7963285 |
| 22q11.2DS | RANBP1 | Deletion | chr22:18877787-21462353 | 2,584,567 | 491 | rs2543958 | rs140392 |
| n/a | RANBP1 | Duplication | chr22:18877787-21462353 | 2,584,567 | 491 | rs2543958 | rs140392 |
| Atypical nested 22q11.2 DupS | RANBP1 | Deletion | chr22:18877787-20772598 | 1,894,812 | 335 | rs2543958 | rs738089 |
| n/a | RANBP1 | Deletion | chr22:18877787-20764595 | 1,886,809 | 333 | rs2543958 | rs1013634 |
| n/a | TYMS | Duplication | chr18:551688-2240220 | 1,688,533 | 370 | rs556830 | rs11080927 |
| Atypical nested 22q11.2DS | RANBP1 | Deletion | chr22:18877787-20295420 | 1,417,634 | 322 | rs2543958 | rs4425183 |
| Atypical nested 22q11.2DS | RANBP1 | Deletion | chr22:18877787-20295420 | 1,417,634 | 322 | rs2543958 | rs4425183 |
| n/a | TJP1 | Duplication | chr15:29429088-30366247 | 937,160 | 206 | rs1877910 | rs10152753 |
| 47XX+DIC | TJP1 | Duplication | chr15:29429088-30329208 | 900,121 | 200 | rs1877910 | rs11070252 |
| n/a | ALDOA | Deletion | chr16:29647342-30177807 | 530,466 | 47 | rs9926100 | rs7202714 |
| n/a | FLNA | Deletion | chrX:153536119-153829693 | 293,575 | 20 | rs17336718 | rs7879049 |
| n/a | CA8 | Duplication | chr8:60964453-61231164 | 266,712 | 58 | rs16925610 | rs10504308 |
| n/a | PDE1C | Duplication | chr7:32241867-32423399 | 181,533 | 61 | rs1860220 | rs215720 |
| n/a | PRDX1 | Deletion | chr1:45936351-46085639 | 149,289 | 16 | rs4660858 | rs2275084 |
| n/a | PLCB3 | Deletion | chr11:63907079-64027888 | 120,810 | 19 | rs7112960 | rs915987 |
| n/a | GRM7 | Deletion | chr3:7078179-7169453 | 91,275 | 35 | rs11713183 | rs1353828 |
| n/a | GRB7 ERBB2 | Duplication | chr17:37834541-37922259 | 87,719 | 3 | rs12150298 | rs907092 |
| n/a | PSMD1 | Deletion | chr2:231940960-232025284 | 84,325 | 7 | rs1472959 | rs6437002 |
| n/a | ACAT1 | Duplication | chr11:107956771-108034124 | 77,354 | 15 | rs11212509 | rs12787445 |
| n/a | PIK3CA | Deletion | chr3:178833722-178886609 | 52,888 | 5 | rs1976765 | rs7641889 |
| n/a | GRIK1 | Deletion | chr21:31289931-31340248 | 50,318 | 11 | rs460583 | rs464809 |
| n/a | PSMD11 | Deletion | chr17:30775761-30812845 | 37,085 | 4 | rs9889352 | rs756785 |
| n/a | ECHS1 | Duplication | chr10:135141572-135178611 | 37,040 | 5 | rs2298122 | rs10857706 |
| n/a | STX12 | Deletion | chr1:28071836-28107059 | 35,224 | 7 | rs1569432 | rs7556606 |
| n/a | TBCA | Deletion | chr5:77008253-77040872 | 32,620 | 5 | rs2652210 | rs352580 |
| n/a | ARL15 | Deletion | chr5:53368171-53399217 | 31,047 | 15 | rs16882121 | rs1703368 |
| n/a | ACCN1 | Deletion | chr17:32224491-32250107 | 25,617 | 10 | rs2637348 | rs902557 |
| n/a | GNB2L1 | Duplication | chr5:180665490-180690937 | 25,448 | 6 | rs2546422 | rs1279912 |
| n/a | HOMER1 | Deletion | chr5:78661854-78673236 | 11,383 | 5 | rs7732902 | rs12187625 |
| n/a | PRKCA | Deletion | chr17:64295321-64305161 | 9,841 | 5 | rs11867898 | rs9901804 |
| n/a | IQGAP2 | Deletion | chr5:75795407-75805105 | 9,699 | 4 | rs7727095 | rs6453227 |
| n/a | RYR2 | Deletion | chr1:237273380-237279140 | 5,761 | 4 | rs12033905 | rs2490389 |
| n/a | RYR2 | Deletion | chr1:237273380-237279140 | 5,761 | 4 | rs12033905 | rs2490389 |
| n/a | ITPR1 | Deletion | chr3:4645916-4651501 | 5,586 | 3 | rs4684427 | rs13327131 |
| n/a | GRM8 | Deletion | chr7:126775198-126779956 | 4,759 | 5 | rs2040502 | rs11563492 |
| n/a | BDKRB2 | Deletion | chr14:96683973-96688608 | 4,636 | 5 | rs945040 | rs8013400 |
| n/a | BDKRB2 | Deletion | chr14:96684770-96688608 | 3,839 | 3 | rs4905466 | rs8013400 |
| n/a | NMI | Deletion | chr2:152143524-152146672 | 3,149 | 3 | rs13383563 | rs2278089 |
| n/a | GRM5 | Deletion | chr11:88376801-88379332 | 2,532 | 4 | rs1354411 | rs11020772 |
| n/a | GRM5 | Duplication | chr11:88533414-88533632 | 219 | 3 | rs10501688 | rs1903841 |
| n/a | GRM5 | Duplication | chr11:88533414-88533632 | 219 | 3 | rs10501688 | rs1903841 |
