# Supplementary material for: The Role of mGluR Copy Number Variation in Genetic and Environmental Forms of Syndromic Autism Spectrum Disorder
Source: Sci Rep. 2016 Jan 19;6:19372. doi: 10.1038/srep19372 (PMC4726047; doi:10.1038/srep19372)
Supplement: Supplementary Table 2 [file srep19372-s3.doc]

**The Role of *mGluR* Copy Number Variation in Genetic and Environmental Forms of Syndromic Autism Spectrum Disorder**

**Tara L. Wenger^1, 2^, Charlly Kao^2^, Donna M. McDonald-McGinn, M.S.^2^, Elaine H. Zackai^2^, Alice Bailey^2^, Robert T. Schultz^2^, Bernice E. Morrow^3^, Beverly S. Emanuel^2^, Hakon Hakonarson^2*^**

^1^Seattle Children’s Hospital, Department of Pediatrics, Seattle, WA 98105 USA

^2^Children’s Hospital of Philadelphia, Department of Pediatrics, Philadelphia, PA 19104 USA

^3^Albert Einstein College of Medicine, Department of Genetics, Bronx, NY 10461 USA

*hakonarson@email.chop.edu

**Supplementary Table 2.** Birth defects seen in Fetal Valproate Syndrome and Thalidomide Embryopathy are all reported in children with 22q11.2 Deletion Syndrome

| Birth Defect | Fetal Valproate Syndrome | Thalidomide Embryopathy | 22q11.2 Deletion Syndrome |
| --- | --- | --- | --- |
| Autism | + | + | + |
| Cleft lip/palate | + | + | + |
| Congenital heart disease | + | + | + |
| Craniosynostosis | + | - | + |
| External Ear anomalies | + | + | + |
| Gastrointestinal tract abnormalities (e.g. atresias) | + | + | + |
| Genitourinary tract anomalies (e.g. renal anomalies) | + | + | + |
| Hypospadias | + | + | + |
| Neural tube defects | + | + | + |
| Ocular anomalies (structural and ocular motility defects) | + | + | + |
| Phocomelia | + | + | + |
| Polydactyly | + | + | + |
| Radial ray defects | + | + | + |
| Vertebral anomalies | + | + | + |
